# The efficacy of controlled stepped intracranial decompression surgery in patients with craniocerebral injury

**DOI:** 10.3389/fneur.2025.1574036

**Published:** 2025-05-15

**Authors:** Xiaobin Huang, Cheng Yu, Xiaoyue Liao, Baizhuo Dong, Jun Zheng, Shanchi Zhang

**Affiliations:** The Second People’s Hospital of Quzhou, Zhejiang, China

**Keywords:** craniocerebral injury, controlled stepwise intracranial decompression, standard large bone flap decompression, efficacy, surgery

## Abstract

**Background:**

Head injuries are frequently the result of high-energy trauma, which is often severe and has a high mortality rate.

**Methods:**

This retrospective study included 78 patients with severe traumatic brain injury treated from January 2021 to January 2023. Patients were divided into two groups: a control group (*n* = 33) treated with standard large bone flap decompression, and a research group (*n* = 45) treated with controlled stepwise intracranial decompression. Surgical parameters, treatment efficacy, complications, neurological function, and serum biomarkers (IL-6, CRP, NSE) were compared. Multivariate logistic regression was adjusted for confounders received.

**Results:**

The research group had significantly shorter decompression initiation times, reduced operation durations, and less intraoperative blood loss (*p* < 0.05). The effective treatment rate was higher in the research group (80.0% vs. 57.6%, *p* < 0.05). After treatment, both groups showed improvements in NFD and GCS scores, with more significant improvement in the research group (*p* < 0.01). Inflammatory markers (IL-6, CRP, NSE) decreased post-treatment in both groups, with significantly lower levels in the research group (*p* < 0.01). The complication rate was markedly lower in the research group (8.9% vs. 30.3%, *p* < 0.05). Multivariate analysis confirmed that stepwise decompression was associated with higher clinical efficacy (aOR = 3.20, 95% CI: 1.24–8.28, *p* = 0.016) and fewer complications (aOR = 0.24, 95% CI: 0.07–0.82, *p* = 0.022). treatment, and NSE levels of the two groups were less than those after therapy (*p* < 0.05); and the blood IL-6, CRP, and NSE levels of the research group after treatment were greater than those of the control group.

**Conclusion:**

Controlled stepped intracranial decompression surgery could effectively shorten the operation time of sufferers with severe craniocerebral injury, reduce intraoperative blood loss, improve clinical treatment effects, improve patient prognosis, and promote neurological recovery.

## 1 Introduction

A head injury is a common neurosurgical problem. It is typically precipitated by high-energy trauma, which is grave and carries a high mortality rate. Given that the majority of patients will present with varying degrees of neurological impairment, the disability rate is also significantly elevated. Consequently, the clinical prognosis is poor (1, 2). It is estimated that between 15 and 20% of patients with severe craniocerebral injuries are accompanied by brain edema or acute intracranial hypertension (3). The effect of conservative treatment is not ideal. Therefore, these patients are often treated with craniotomy and decompression surgery in clinical practice, which can effectively reduce intracranial hypertension (4). Standard craniocerebral decompression is one of the common procedures for treating craniocerebral injuries. It could obviously have an improvement on the sufferer’s condition and save their lives (5). Nevertheless, there have been reports indicating that the intracranial pressure in patients with severe craniocerebral injuries may decline excessively rapidly during surgical procedures. This could potentially result in the sudden release of the pressure tamponade effect, thereby increasing the risk of complications such as large-scale cerebral infarction and acute encephalocele during the operation. Such complications could significantly impact the patient’s life, health, and safety (6). Therefore, it is particularly important to explore a method that can gradually control the reduction of intracranial pressure and reduce the risk of complications to improve the prognosis of sufferers with craniocerebral injury. Controlled stepped intracranial decompression surgery is a modified procedure based on standard cranial decompression, which can significantly control the decompression effect of intracranial pressure and prevent damage to brain tissue due to the tamponade effect caused by rapid decompression (7–9). This research aims to explore the impact of controlled stepwise intracranial decompression surgery on the clinical efficacy of patients with craniocerebral injury. The objective is to provide new ideas for clinical treatment guidance and improve patient prognosis.

## 2 Materials and methods

### 2.1 Normal information

A total of 78 patients with craniocerebral injury admitted to the Department of Critical Medicine, the Second People’s Hospital of Quzhou City, from January 2021 to January 2023, were consecutively enrolled as research subjects. Group assignment was based on the chronological order of admission and clinical equipoise: Control group (*n* = 33): Patients admitted from January 2021 to June 2022 received standard large bone flap decompression surgery; Research group (*n* = 45): Patients admitted from July 2022 to January 2023 underwent controlled stepwise intracranial decompression surgery. The allocation was non-randomized but ensured baseline comparability through propensity score matching (PSM). Matching variables included age, sex, Glasgow Coma Scale (GCS) score, intracranial hematoma volume, and injury time. Post-matching analysis confirmed no statistically significant differences in baseline characteristics between the two groups (*p* > 0.05, Table 1) methods. Their clinical outcomes were retrospectively analyzed. The specific baseline comparison is as follows: the control group had 20 men and 13 women, and the mean age was (44.39 ± 11.70) years, with 17 instances of unilateral pupil dilation, six instances with bilateral pupil dilation, intracranial hematoma volume in mL, and average intracranial hematoma volume. (67.21 ± 8.37) mL, the GCS score was (4.21 ± 0.60) and the time of injury was (7.21 ± 1.37) days; in the research group, there were 27 men and 18 women, and the mean age was (44.76 ± 11.65) years. It had 25 instances of unilateral pupil dilation and eight instances of bilateral pupil dilation. The average intracranial hematoma volume was (67.24 ± 8.29) mL, the GCS score was 4.36 ± 0.50, and the time of injury was (7.24 ± 1.29) days. There was no statistically obvious distinction in the general data of the two groups after analysis with statistical software (*p* > 0.05, Table 1). This study has been approved by the Ethics Committee of our hospital [Approval 2023 Ethics (38)].

**Table 1:** General data analysis of the two groups [*n* (%), ($\bar{x}\pm s$)].

Group	*n*	Gender (male/female)	Age (years)	Mydriasis (unilateral/bilateral)	Intracranial hematoma volume (mL)	GCS score (points)	Time of injury (days)
Control group	33	20/13	44.39 ± 11.70	17/6	67.21 ± 8.37	4.21 ± 0.60	7.21 ± 1.37
Research group	45	27/18	44.76 ± 11.65	25/8	67.24 ± 8.29	4.36 ± 0.50	7.24 ± 1.29
*χ*^2^/*t*		0.003	−0.138	0.002	−0.016	−1.202	−0.099
*p* value		0.957	0.890	0.962	0.987	0.233	0.922

### 2.2 Inclusion and exclusion criteria

Inclusion criteria: (1) All sufferers meet the clinical symptoms of craniocerebral injury; (2) All sufferers are diagnosed with craniocerebral injury through imaging examinations such as CT or MRI; (3) All sufferers have complete clinical data such as blood routine and laboratory tests; (4) The Glasgow Coma Scale (GCS) was 3–8 points; (5) The neurological deficit (NFD) score (10) was 31–45 points; (6) All sufferers received decompression surgery within 2 h after admission; (7) In accordance with the Declaration of Helsinki and approved by our Ethics Committee [Approval No: 2023 Ethics (38)].

**Figure 1:**
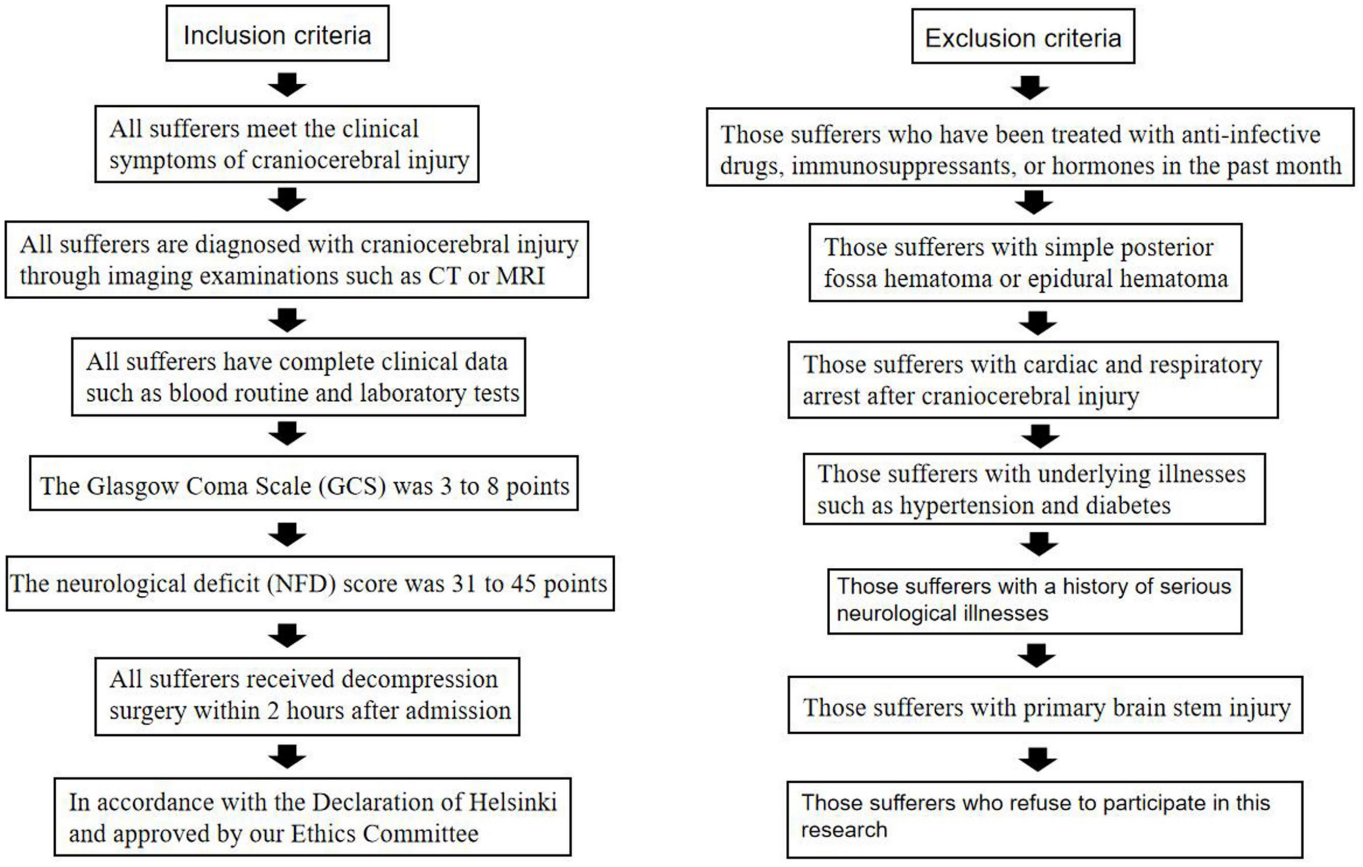
The schematic diagram of the process of inclusion and exclusion criteria.

Exclusion criteria: (1) Those sufferers who have been treated with anti-infective drugs, immunosuppressants, or hormones in the past month; (2) Those sufferers with simple posterior fossa hematoma or epidural hematoma; (3) Those sufferers with cardiac and respiratory arrest after craniocerebral injury; (4) Those sufferers with underlying illnesses such as hypertension and diabetes; (5) Those sufferers with a history of serious neurological illnesses; (6) Those sufferers with primary brain stem injury; (7) Those sufferers who refuse to participate in this research (Figure 1).

### 2.3 Method

The sufferers in the control group received standard craniectomy and general anesthesia with tracheal intubation. An incision is made in the frontotemporal scalp according to the location of the lesion detected by imaging examination, the bone flap is removed, and the dura mater is quickly cut radially to remove the patient’s intracranial hematoma and inactivated brain tissue to quickly reduce intracranial pressure. The research group received controlled stepwise intracranial decompression surgery, using the CODMAN ventricular intracranial pressure monitor (provided by Shanghai Jumu Medical Instruments Co., Ltd.), placing its probe on the anterior corner of the lateral ventricle on the contralateral side of the surgery, observing and recording the patient’s initial intracranial pressure. An incision was made in the frontotemporal scalp according to the location of the lesion detected by imaging examination. Then a hole was drilled at the most severe position of the patient’s brain injury to expand it into a 2 cm × 2 cm bone window. The dura mater was cut and further reduced intracranial pressure gradually and slowly according to the monitoring results of the intracranial pressure monitor. The bone flap was quickly removed, and the bone window was further expanded to 12 cm × 15 cm. The dura mater was incised sequentially to slowly release cerebrospinal fluid and hematoma, and gradually reduce the patient’s intracranial pressure. The radioactive incision is made into the dura mater, and then the patient’s intracranial hematoma and inactivated brain tissue are cleaned. If the brain swelling is obvious, the brain tissue in the non-functional area may be removed as appropriate to achieve decompression. When the patient’s intracranial pressure continues to be lower than 20 mmHg, the artificial dural repair is performed to reduce tension and repair. The dura mater is sutured, a drainage tube is placed in the dura mater, and the skull is closed. After the operation, both groups of patients were given symptomatic treatment such as oxygen inhalation, hemostasis, and prevention of vasospasm, and blood routine, urine routine, coagulation function, and other indicators were regularly reviewed. All patients were followed up for 3 months.

### 2.4 Observation indicators

(1) Surgical indicators: Time to start decompression (defined as time from skin incision to initial pressure relief), intraoperative blood loss (measured by suction volume + weight of surgical gauze), and total operation time (from incision closure) were recorded by the surgical team. (2) Neurological deficit and prognosis: Neurological function was assessed using the NFD score and GCS at three time points: preoperatively, 24 h postoperatively, and 7 days postoperatively. Evaluations were performed independently by two neurologists blinded to group allocation. Discrepancies >10% in scores were resolved by a third senior neurologist. Inter-rater reliability was confirmed (Cohen’s *κ* = 0.85). (3) Clinical efficacy: Excellent: the patient’s NFD score 1 week after treatment is reduced by 91–100% compared with before treatment; good: the patient’s NFD score 1 week after treatment is reduced by 46–90% compared with before treatment; medium: the patient’s NFD score 1 week after treatment is reduced by 18–45% in comparison with that at pre-therapy; poor: the patient’s NFD score 1 week after treatment decreased by 18% or increased compared with before treatment. Significant efficiency (%) = (number of excellent cases + number of good cases)/total number of observed cases × 100% (11). To minimize bias, efficacy adjudication was conducted by a committee comprising a neurosurgeon, rehabilitation specialist, and statistician, all blinded to surgical allocation. (4) Serum interleukin-6 (IL-6), C-reactive protein (CRP), and neuron-specific enolase (NSE) levels: 5 mL of cubital venous blood was drawn from the two groups at pre-therapy and third day after treatment, and placed on the desktop Centrifuge at a speed of 3,000 r/min for 10 min in a high-speed low-temperature centrifuge and take the supernatant. Serum IL-6, CRP, and NSE levels in the research group and control group were detected by enzyme-linked immunosorbent assay (ELISA, Millipore, Billerica, MA, USA; R&D Systems, Minneapolis, MN, USA). Each plate included duplicate standards and internal controls. Coefficients of variation (CV) < 15% were accepted. Assays were performed by laboratory technicians blinded to clinical data. (5) Complications: Postoperative complications were systematically defined and monitored using integrated imaging and clinical criteria. Massive cerebral infarction was defined as ischemic involvement of >1/3 cerebral hemisphere on postoperative CT/MRI accompanied by persistent neurological deficits (e.g., hemiplegia, aphasia) lasting >24 h. Delayed hematoma required radiographic confirmation ≥24 h postoperatively with a volume ≥30 mL, necessitating surgical intervention. Acute encephalocele was diagnosed by imaging evidence (midline shift >5 mm or basal cistern obliteration) combined with clinical signs (anisocoria, GCS decline ≥2 points, or respiratory instability). All patients underwent standardized CT surveillance at 6 h, 24 h, 72 h, and 1 month postoperatively, with additional scans triggered by neurological deterioration (GCS decrease ≥2 points or new focal deficits). Clinical monitoring included hourly pupil/limb assessments during the initial 48 h, daily blinded GCS and NFD evaluations by neurologists, and continuous intracranial pressure monitoring for 72 h in patients with GCS ≤ 8. An independent adjudication committee comprising neurosurgeons, radiologists, and intensivists reviewed all potential complications through blinded assessment, resolving discrepancies via consensus. Follow-up encompassed two phases: in-hospital active surveillance (days 0–14) and outpatient tracking (days 15–30), the latter involving structured telephone interviews and repeat CT scans for high-risk patients to ensure comprehensive complication detection.

### 2.5 Statistical methods

Data analysis was performed using SPSS 21.0 software (Armonk, NY, USA: International Business Machines Corporation). Anderson-darling was used to test whether the data obeyed normality. Count data were expressed by *n* (%). Pairwise comparisons were made by the *x*^2^ test. Measurement data were represented by ($\bar{x}\pm s$). Pairwise comparisons were made using an independent samples *t-test*. *p* < 0.05 was considered a statistically significant distinction.

## 3 Results

### 3.1 Surgical indicators

Compared to the control group, the time to start decompression, intraoperative blood loss, and operation time in the research group obviously decreased (*p* < 0.05) (Table 2).

**Table 2:** The surgical indicators compared between the two groups ($\bar{x}\pm s$).

Group	*n*	Start decompression time (min)	Intraoperative blood loss (mL)	Operation time (h)
Control group	33	28.64 ± 7.26	122.09 ± 15.51	2.00 ± 0.66
Research group	45	11.98 ± 4.21	104.16 ± 17.23	1.56 ± 0.62
*t*		14.736	2.438	3.031
*p* value		0.000*	0.017*	0.003*

p* indicates comparison with that at pre-therapy, *p* < 0.05.

### 3.2 Clinical efficacy

There were 12 excellent cases and 24 good cases in the research group, with a marked efficiency of 80.00% (36/45). There were seven excellent cases and 12 good instances in the control group, with a marked efficiency of 57.58% (19/33). There was an obvious distinction between the research group and the control group. The difference in efficiency is statistically significant (*p* < 0.05) (Table 3).

**Table 3:** The total effective rate of treatment compared between the two groups [*n* (%)].

Group	*n*	Excellent	Good	Middle	Difference	Significant efficiency
Control group	33	7 (21.21)	12 (36.36)	11 (33.33)	3 (9.09)	19 (57.58)
Research group	45	12 (26.67)	24 (53.33)	7 (15.56)	2 (4.44)	36 (80.00)
*χ* ^2^						4.604
*p* value						0.032*

p* indicates comparison with that at pre-therapy, *p* < 0.05.

### 3.3 Neurological deficit and prognosis

After therapy, the NFD scores and GCS points of the two groups were decreased compared with those at pre-therapy (*p* < 0.05); and the NFD scores and GCS points of the research group after therapy were reduced in comparison to the control group (*p* < 0.01) (Table 4).

**Table 4:** The NFD points and GCS points compared between the two groups ($\bar{x}\pm s$).

Group	*n*	NFD score		GCS score	
		Before therapy	After therapy	Before therapy	After therapy
Control group	33	38.67 ± 4.24	18.70 ± 3.28	4.21 ± 0.60	6.21 ± 0.60
Research group	45	38.12 ± 4.31	11.85 ± 2.94	4.36 ± 0.50	7.36 ± 0.50
*t*		0.593	9.615	−1.202	−9.218
*p* value		0.555	0.000*	0.233	0.000*

p* indicates comparison with that at pre-therapy, *p* < 0.05.

### 3.4 IL-6, CRP, and NSE levels

After therapy, the serum IL-6, CRP, and NSE levels of the two groups were obviously less than those after therapy (*p* < 0.05); and the blood IL-6, CRP, and NSE levels of the research group at post-therapy were obviously greater than the control group (*p* < 0.01) (Table 5).

**Table 5:** The IL-6, CRP and NSE levels compared between the two groups ($\bar{x}\pm s$).

Group	Time	NSE (μg/L)	IL-6 (ng/L)	CRP (mg/L)
Control group (*n* = 33)	Before treatment	24.66 ± 3.45	33.57 ± 4.98	56.28 ± 6.59
	After treatment	16.53 ± 2.77	25.68 ± 3.63	29.25 ± 3.66
*t*		10.556	7.355	20.599
*p* value		0.000	0.000	0.000
Research group (*n* = 45)	Before treatment	24.98 ± 3.61	33.86 ± 5.07	55.76 ± 6.48
	After treatment	11.35 ± 2.32	18.53 ± 3.15	20.54 ± 3.35
*t*		5.572	17.229	32.388
*p* value		0.000*	0.000*	0.000*

p* indicates comparison with control group at post-therapy, *p* < 0.05.

### 3.5 Complications

In the research, two instances of large-area cerebral infarction, one case of delayed hematoma, and one instance of acute encephalocele occurred. The total frequency rate of complications was 8.89% (4/45). In the control group, three instances of large-area cerebral infarction occurred. There were three cases of hematoma and four cases of acute encephalocele. The total frequency ratio of complications was 30.30% (10/33). The total frequency ratio of complications in the research group was statistically significant compared with the control group (*p* < 0.05) (Table 6).

**Table 6:** The complication rates compared between the two groups [*n* (%)].

Group	*n*	Massive cerebral infarction	Delayed hematoma	Acute encephalocele	Overall incidence
Control Group	33	3 (9.09)	3 (9.09)	4 (12.12)	10 (30.30)
Research Group	45	2 (4.44)	1 (2.22)	1 (2.22)	4 (8.89)
*χ* ^2^		4.457	5.538	6.277	5.928
*p* value		0.033	0.019	0.012	0.015*

p* indicates comparison with control group at post-therapy, *p* < 0.05.

### 3.6 Multivariate analysis of clinical efficacy and complications

Multivariate logistic regression was performed to adjust for potential confounders for the primary outcomes (clinical efficacy and complications). Covariates included age, sex, preoperative GCS score, intracranial hematoma volume, and injury time. Variables were assigned in Table 7.

**Table 7:** Variable assignment table.

Variable	Assignment
Group (Research vs. Control)	0 = Control, 1 = Research
Sex	0 = Female, 1 = Male
Unilateral pupil dilation	0 = No, 1 = Yes
Age	Continuous (years)
GCS score	Continuous (points)
Intracranial hematoma volume	Continuous (mL)
Injury time	Continuous (days)

After adjusting for covariates, the research group showed a higher likelihood of achieving significant efficacy compared to the control group (OR = 3.20, 95% CI: 1.24–8.28, *p* = 0.016). Higher preoperative GCS scores were associated with improved efficacy (OR = 1.58, 95% CI: 1.02–2.45, *p* = 0.042). Other variables (age, sex, hematoma volume) did not reach statistical significance (Table 8). The research group had lower risk of complications compared to the control group (OR = 0.24, 95% CI: 0.07–0.82, *p* = 0.022). Larger hematoma volume was independently associated with increased complication risk (OR = 1.08 per mL, 95% CI: 1.01–1.15, *p* = 0.031) (Table 8).

**Table 8:** Multivariate logistic regression analysis of clinical efficacy and complications.

Outcome	Variable	OR (95% CI)	*p* value
Efficacy	Group (Research)	3.20 (1.24–8.28)	0.016
	Age	0.98 (0.94–1.03)	0.420
	Sex (Male)	1.12 (0.45–2.81)	0.810
	GCS score	1.58 (1.02–2.45)	0.042
	Hematoma volume	0.99 (0.94–1.05)	0.790
	Injury time	1.02 (0.75–1.39)	0.890
Complications	Group (Research)	0.24 (0.07–0.82)	0.022
	Age	1.01 (0.96–1.06)	0.720
	Sex (Male)	1.45 (0.48–4.37)	0.510
	GCS score	0.87 (0.52–1.45)	0.590
	Hematoma volume	1.08 (1.01–1.15)	0.031
	Injury time	0.95 (0.65–1.38)	0.780

OR, Odds Ratio; CI, Confidence Interval.

## 4 Discussion

Severe craniocerebral injury can cause clinical symptoms, for example, disturbance of consciousness, nausea, and headache in patients, and severe cases may even be life-threatening. Timely and effective reduction of intracranial pressure is the basic principle of treating patients with craniocerebral injury (12). Commonly used treatment methods for craniocerebral injury, combining symptomatic treatment with surgery, can significantly improve patients’ clinical symptoms, relieve high intracranial pressure, and reduce mortality (13, 14). Flynn et al. (15) found that 17 sufferers with traumatic brain injury were treated with standard craniectomy. The patients’ intracranial hematoma and necrotic brain tissue were removed through craniotomy, and the impact of the hematoma on the intracranial tissue was relieved. The resulting physiological and pathological damage saved the patient’s life. Rapidly clearing intracranial hematoma and necrotic brain tissue during the sufferer’s surgery could easily cause a rapid reduction in intracranial pressure, leading to pressure tamponade effects, as well as ischemia–reperfusion injury, which could lead to damage to blood vessels and neural tissues in the brain and affect the sufferer’s clinical prognosis (16). Research by Honeybul et al. (17) showed that controlled stepwise intracranial decompression has reduction. It can regulate the rate of intracranial pressure reduction and plays a vital role in treating severe craniocerebral injury. Function and clinical efficacy are ideal. This study retrospectively analyzed the clinical effect of controlled cranial decompression in different patients with craniocerebral injury and how to improve the prognosis of patients, and provided a new idea. This study found that the effective rate of the research group was 80.00% (36/45), which was greater than the control group, which was 57.58% (19/33). The total incidence of complications was 8.89% (4/45), which was greater than that of the control group. The incidence rate was 30.30% (10/33), obviously less. The results of neurological deficit and prognosis assessment showed that post-therapy, the NFD scores and GCS points of the two groups were reduced compared with those at pre-therapy; however, after treatment, the NFD scores and GCS points of the research group were less than the control group. There was a decrease. It shows that controlled stepped intracranial decompression surgery could effectively have an improvement on the clinical therapy effect of sufferers with craniocerebral injury, decrease the incidence of complications, promote the recovery of patients’ neurological function, and improve the prognosis.

The observed reductions in serum IL-6, CRP, and NSE levels post-treatment, particularly the more pronounced decreases in the research group, underscore the clinical relevance of controlled stepped decompression. NSE, a glycolytic enzyme enriched in neuronal and neuroendocrine tissues, serves as a sensitive biomarker of neuronal injury; elevated serum levels reflect axonal damage and correlate with poor neurological outcomes in TBI (18–20). The significant reduction in NSE levels observed in the research group suggests attenuated neuronal injury, likely attributable to gradual intracranial pressure (ICP) normalization, which minimizes mechanical stress on vulnerable neural structures (21, 22). Similarly, IL-6, a key mediator of neuroinflammation, exacerbates secondary brain injury by promoting blood–brain barrier disruption and leukocyte infiltration (23, 24). The marked decline in IL-6 levels in the research group indicates mitigated neuroinflammatory cascades, aligning with their superior neurological recovery (as evidenced by NFD and GCS improvements). CRP, a systemic inflammatory marker, not only reflects acute-phase responses but also predicts long-term cognitive deficits in TBI (25, 26). The sharper CRP reduction in the research group highlights enhanced systemic inflammatory control, potentially reducing secondary insults such as edema and ischemia–reperfusion injury. Collectively, these biomarker trends suggest that controlled decompression modulates neuroinflammatory pathways and preserves microvascular integrity, thereby fostering neurorepair and functional recovery.

This may be because although standard craniectomy has a significant effect on removing intracranial hematoma and necrotic brain tissue, this surgery has a relatively fast removal rate and can directly and quickly remove brain tissue in the temporal lobe and other non-functional areas. This causes the patient’s intracranial pressure to drop too quickly, resulting in the formation of a pressure tamponade effect, which triggers cerebral ischemia–reperfusion injury. Local inflammatory reactions occur and spread to deep brain tissues, resulting in cerebral vasospasm and further aggravation of intracranial hematoma and complications. Damage to nerve tissue, in severe cases even beyond the tolerance range of blood vessels and barriers, causing damage to the already damaged blood vessels and barriers again, or even bleeding, and leading to a greatly increased risk of complications such as acute encephalocele, cerebral infarction, etc., affecting patients’ recovery (27–30). Controlled stepped intracranial decompression surgery uses step-by-step decompression to treat patients with intracranial hematoma. This surgery can control the rate of intracranial pressure drop within a relatively gentle and safe range, which is beneficial to the brain. The internal blood vessels, plate barriers, etc. gradually and slowly adapt to the reduction rate of intracranial pressure, which can prevent the body from inducing post-ischemic reperfusion injury and local inflammatory reaction (31, 32); In addition, this surgery can also improve the patient’s cranial pressure. Internal inflammatory state, inhibiting the secretion of inflammatory factors such as IL-6 and CRP, which is beneficial to reducing damage to brain tissue and providing favorable conditions for patients to recover after surgery (33, 34).

The innovation of this study lies in the integration of inflammatory biomarkers (IL-6, CRP, NSE), providing mechanistic insights into neuroprotection and systemic inflammatory modulation, strengthening the study’s translational relevance for traumatic brain injury management. This study has several limitations that warrant consideration. First, the exclusion of patients with common comorbidities such as hypertension and diabetes, while intended to isolate the surgical effects, may introduce selection bias and limit the generalizability of findings to broader TBI populations with complex medical profiles. Second, the single-center retrospective design inherently restricts causal inference and increases susceptibility to unmeasured confounders. Third, the 3-month follow-up period precludes assessment of long-term functional outcomes or delayed complications, such as chronic hydrocephalus or post-traumatic epilepsy. While the observed short-term benefits in neurological recovery and inflammatory modulation are promising, their durability beyond the study timeframe remains undetermined. Future multicenter prospective studies with extended follow-up durations are needed to validate these findings and evaluate their clinical sustainability.

## 5 Conclusion

In summary, controlled stepped intracranial decompression surgery can effectively shorten the operation time of sufferers with serious craniocerebral injury, reducing intraoperative blood loss to have the improvement on clinical therapy effects and sufferer’s prognosis, promote neurological recovery, and reduce the risk of complications.

## Data Availability

The original contributions presented in the study are included in the article/supplementary material, further inquiries can be directed to the corresponding authors.
